# Bilateral Apical Congenital Pulmonary Airway Malformation Presenting With Spontaneous Pneumothorax in an Adolescent: A Case Report

**DOI:** 10.7759/cureus.109394

**Published:** 2026-05-21

**Authors:** Alhan Fernando Castillo Valencia, Jorge Alan Perez Liñan, Regina Gallardo Santiago, Oswaldo Sánchez Alvarez, Olga Álvarez Kraus

**Affiliations:** 1 General Surgery, Hospital Regional Universitario de Colima, Colima, MEX; 2 Pediatric Surgery, Hospital Regional Universitario de Colima, Colima, MEX; 3 Surgery, Centro Médico Nacional de Occidente IMSS, Guadalajara, MEX; 4 Pathology, Hospital Regional Universitario de Colima, Colima, MEX

**Keywords:** adolescent, bilateral apical lesions, case report, congenital cystic adenomatoid malformation, congenital pulmonary airway malformation, cystic lung lesion, histopathology, segmentectomy, spontaneous pneumothorax, stocker type 2

## Abstract

Congenital pulmonary airway malformation (CPAM), previously termed congenital cystic adenomatoid malformation (CCAM), is a rare developmental anomaly of the lower respiratory tract that is most commonly diagnosed prenatally or during early infancy. Presentation in adolescence is uncommon, particularly when associated with bilateral lesions and pneumothorax. We report the case of a 14-year-old female with no relevant past medical history who presented with sudden-onset progressive dyspnea and was found to have an approximately 50% right-sided pneumothorax. Contrast-enhanced chest computed tomography demonstrated bilateral apical cystic lesions initially interpreted as pneumatoceles, measuring 25 × 10 mm in the right lung apex and 10 × 18 mm in the left lung apex. After initial pleural drainage, the patient underwent right apical wedge resection, followed one week later by left-sided wedge resection. Histopathological examination of both specimens revealed pulmonary tissue with congested vessels, focal alveolar collapse, and multiple cystic spaces of variable size with thin fibrous walls and focal simple cuboidal epithelial lining. CD31 immunostaining was negative in the cyst lining, and Ziehl-Neelsen staining was negative. The overall findings were consistent with Stocker type 2 CPAM. This case highlights an unusual adolescent presentation of bilateral apical CPAM complicated by pneumothorax and underscores the importance of histopathologic confirmation when imaging findings are atypical or inconclusive.

## Introduction

Congenital pulmonary airway malformation (CPAM), previously referred to as congenital cystic adenomatoid malformation (CCAM), is a rare developmental anomaly of the lower respiratory tract characterized by abnormal proliferation of terminal bronchioles and the formation of cystic lung lesions [1]. This condition arises from disrupted airway morphogenesis during early fetal development and represents one of the most common congenital cystic lung lesions [2].

The estimated incidence of CPAM ranges between 1 in 10,000 and 1 in 35,000 live births, although increasing use of prenatal imaging has led to more frequent detection in recent decades [3]. Currently, antenatal USG is the most common mode of diagnosis, identifying up to 79% of cases before birth, with most lesions presenting as radiologically evident abnormalities at delivery. While some lesions may regress spontaneously, the majority persist and require postnatal evaluation [2,3].

CPAM is classified according to the Stocker classification system into multiple subtypes based on histopathological characteristics, with type I being the most prevalent and typically associated with a more favorable prognosis [1,2]. Clinically, CPAM most commonly presents in the neonatal period with respiratory distress or later in childhood with recurrent pulmonary infections. However, a subset of patients may remain asymptomatic for years, leading to delayed diagnosis in adolescence or adulthood [3,4].

Although uncommon, CPAM may present with acute complications such as spontaneous pneumothorax, particularly in older patients with cystic lesions prone to rupture [4]. This atypical presentation can pose a diagnostic challenge and may delay appropriate management. Furthermore, CPAM has been associated with potential malignant transformation, including bronchioloalveolar carcinoma/adenocarcinoma, reinforcing the importance of early recognition and treatment [5,6].

The management of CPAM remains controversial, particularly in asymptomatic patients. Evidence from systematic reviews indicates that approximately 16-24% of initially asymptomatic patients develop symptoms requiring surgical intervention during conservative management [5]. A meta-analysis demonstrated that elective surgical resection is associated with significantly lower complication rates (10.0%) compared to surgery performed after symptom development (31.8%), with an OR of 4.59 (95% CI 1.40-15.11, P < 0.01) [7]. However, recent prospective data suggest that conservative observation is safe and feasible for asymptomatic infants, with only 15% requiring surgery due to symptoms within the first two years of life [8]. Consequently, the optimal management strategy continues to be debated, with some authors advocating for early surgical intervention to prevent complications, while others support close monitoring with selective surgery based on symptom development.

We report a rare case of bilateral apical CPAM presenting as spontaneous pneumothorax in an adolescent patient, highlighting an unusual clinical presentation and emphasizing the importance of considering congenital lung malformations in the differential diagnosis of spontaneous pneumothorax in young individuals. This case contributes to the limited literature on bilateral CPAM, a particularly rare variant reported in fewer than 2% of CPAM cases, and poses unique diagnostic and therapeutic challenges. Furthermore, the apical location of the lesions represents an atypical anatomical distribution, as CPAM most commonly affects the lower lobes.

The documentation of this case is clinically relevant for three reasons: first, it expands the phenotypic spectrum of CPAM presentations that clinicians should consider when evaluating young patients with spontaneous pneumothorax; second, it provides insight into the natural history of undetected bilateral lesions that may remain asymptomatic until adolescence; and third, it raises important questions regarding surveillance strategies and surgical decision-making in patients with bilateral disease, where the risk-benefit analysis of prophylactic resection differs substantially from that in unilateral cases.

## Case presentation

In March 2026, a 14-year-old female with no relevant comorbidities presented to the ED with acute sudden-onset dyspnea of less than 40 minutes’ duration, which began during physical exertion and progressively worsened. On initial evaluation, she showed clinical signs of respiratory distress and findings consistent with pneumothorax. Physical examination revealed absent breath sounds in the right hemithorax, while vesicular breath sounds were preserved on the left side.

A chest radiograph demonstrated an approximately 50% right-sided pneumothorax. A 14-Fr right pleural tube was placed. Based on the patient’s clinical presentation and radiologic findings, the lesions were considered consistent with a congenital cystic pulmonary malformation, specifically CCAM/CPAM, predominantly affecting the right upper lobe apex. Due to the symptomatic presentation complicated by secondary pneumothorax, surgical management was indicated. The patient underwent right wedge resection through open thoracotomy, as video-assisted thoracoscopic surgery (VATS) equipment was not available at our institution. The immediate postoperative course was satisfactory, with a follow-up chest radiograph showing adequate pulmonary re-expansion. After the surgical procedure, the pleural chest tube had serosanguineous drainage of less than 100 mL within 24 hours and was removed 48 hours postoperatively. One week later, a left-sided wedge resection was performed through the same approach, with similar satisfactory postoperative evolution and adequate contralateral pulmonary re-expansion. Similarly, after the second surgical procedure, the pleural chest tube showed serosanguineous drainage of less than 100 mL within 24 hours and was removed 48 hours postoperatively. No postoperative complications occurred. Follow-up evaluations at 7 and 30 days after discharge demonstrated continued clinical improvement.

**Figure 1:**
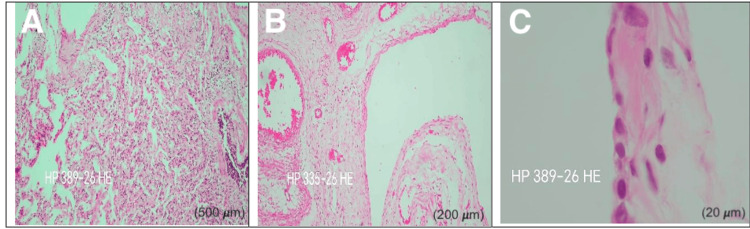
Histopathological examination of the resected lung specimens. (A, B) Hematoxylin and eosin-stained sections show residual lung parenchyma with variably sized cystic spaces and thin fibrous walls. (C) High-power view demonstrates focal simple cuboidal epithelial lining.

Microscopic examination of both specimens revealed residual lung parenchyma with vascular congestion and focal alveolar collapse, in association with multiple variably sized cystic spaces measuring 2 to 12 mm on histologic sections. These spaces were lined by thin fibrous walls; some showed an inner lining of simple cuboidal epithelium, while in others the lining was flattened or not identifiable (Figure 1).

**Figure 2:**
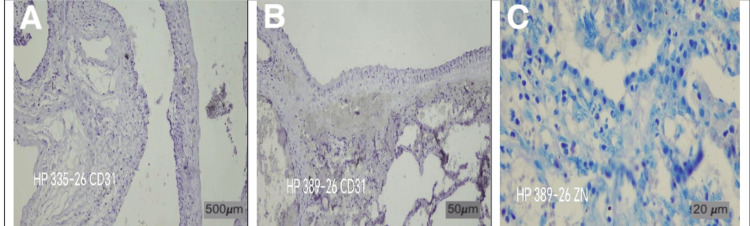
Histopathological examination. (A, B) Cluster of differentiation 31 (CD31) immunostaining is negative in the cyst lining, arguing against a vascular lesion. (C) Ziehl-Neelsen staining is negative for acid-fast bacilli. Overall, the findings are consistent with congenital pulmonary airway malformation/congenital cystic adenomatoid malformation (CPAM/CCAM), Stocker type 2. CPAM: Congenital pulmonary airway malformation; CCAM: Congenital cystic adenomatoid malformation.

To exclude a vascular malformation and mycobacterial infection, ancillary studies were performed. CD31 immunostaining was negative in the cyst lining, arguing against a vascular lesion, and Ziehl-Neelsen staining was negative for acid-fast bacilli. Given that pulmonary vascular lesions may occur in pediatric patients and may be associated with involvement of other organs, CD31 immunostaining was specifically requested; however, no immunoreactivity was identified in the cyst lining. In the context of congenital cystic adenomatoid malformation/congenital pulmonary airway malformation, the presence of small cystic spaces with focal simple cuboidal epithelial lining and areas of ciliary loss was most consistent with Stocker type 2 (Figure 2).

This subtype, together with type 1, is generally considered to have a favorable prognosis. Management was planned, and the patient underwent right wedge resection performed through open thoracotomy according to the previously established treatment plan. One week later, a left-sided wedge resection was also performed.

## Discussion

This case represents an uncommon presentation of CPAM. Most CPAMs are currently identified prenatally or during early infancy, whereas presentation in adolescence is rarely reported [7]. In addition, CPAM usually involves a single lobe; therefore, the presence of bilateral apical lesions in this patient makes the case particularly unusual [8,9]. The clinical presentation was also atypical, as the patient presented with pneumothorax rather than the more commonly reported manifestations of neonatal respiratory distress or recurrent pulmonary infection [10,11]. Another notable feature was the initial CT interpretation of the lesions as pneumatoceles, illustrating how late and atypical presentations may obscure the congenital nature of the disease [12-14]. In this context, the final diagnosis was established only after surgical resection and histopathologic evaluation.

Diagnostic challenge in this case

The main diagnostic challenge in this case was the limited specificity of imaging for distinguishing congenital cystic malformations from acquired cystic lung lesions. On CT, both pneumatoceles and type 2 CPAM may appear as thin-walled cystic lesions, particularly when the clinical setting favors an acquired explanation [13-15]. This limitation is clinically relevant, as prior studies have shown that imaging-pathology concordance in congenital lung malformations is imperfect, even when postnatal CT is available [16,17].

In this patient, histopathology was essential for diagnostic confirmation. The presence of multiple small cystic spaces measuring 2-12 mm, thin fibrous walls, and focal simple cuboidal epithelial lining supported the diagnosis of Stocker type 2 CPAM [18,19]. Areas with flattened or absent epithelial lining may reflect secondary distortion or partial epithelial loss and do not exclude the diagnosis in the appropriate morphologic setting [20]. Ancillary studies further strengthened the interpretation: cluster of differentiation 31 (CD31) negativity in the cyst lining argued against an endothelial-lined vascular lesion, while Ziehl-Neelsen negativity helped exclude acid-fast bacillary infection, including mycobacterial disease, in the context of bilateral apical cystic lesions [20]. Taken together, these findings show that in atypical cases, definitive diagnosis may depend more on pathology than on imaging alone.

Surgical relevance

Surgical treatment was justified in this case because the patient was symptomatic and had already presented with pneumothorax [7-11]. Resection offered two major benefits: it addressed the clinically relevant lesions and provided a definitive tissue diagnosis in a case where imaging findings were not conclusive [13-17]. This dual diagnostic and therapeutic role is particularly important in patients with an unusual age at presentation and nonclassic radiologic findings.

The use of staged bilateral wedge resection also appears reasonable in this setting. Because the lesions were bilateral and limited to the apical segments, a parenchyma-sparing approach allowed treatment while preserving as much functional lung tissue as possible. Although lobectomy remains the traditional standard for many congenital lung malformations, recent literature supports wedge resection in selected patients when anatomically feasible, particularly when preservation of lung parenchyma is desirable [21-25]. In this case, the bilateral distribution made lung preservation especially relevant.

Clinical implications

This case highlights several practical points. First, CPAM can rarely remain undiagnosed until adolescence, especially when symptoms are absent or nonspecific during childhood [9-26]. Second, bilateral apical cystic lesions in an adolescent with pneumothorax should prompt consideration of an underlying congenital pulmonary lesion, even when the initial radiologic impression suggests a more common acquired process. Third, this case underscores the importance of radiologic-pathologic correlation in cystic lung disease, as imaging alone may not reliably distinguish among congenital, infectious, inflammatory, and other cystic entities [13-17].

From a prognostic standpoint, type 2 CPAM is generally regarded as a favorable subtype after complete resection, with a lower documented association with malignancy than type 1 lesions [27]. Nevertheless, the rarity of adolescent presentations makes long-term outcome data in this subgroup limited.

Need for further research

This case also illustrates important gaps in current knowledge. Evidence regarding late and atypical adolescent presentations of CPAM remains limited, as most published data focus on prenatal diagnosis and early childhood management [7-26]. Similarly, more work is needed to improve the radiologic distinction between CPAM and lesions initially interpreted as pneumatoceles or other acquired cystic abnormalities, since diagnostic uncertainty at imaging may directly influence management [13-17].

Another unresolved issue is the optimal surgical strategy for bilateral, anatomically limited lesions. Although parenchyma-sparing resection is increasingly reported, the literature remains insufficient to define the best approach, timing, and long-term outcomes in this specific setting [21-25]. Finally, long-term follow-up data for resected Stocker type 2 lesions diagnosed in adolescence are scarce. Further studies are needed to better define recurrence risk, long-term pulmonary outcomes, and the true prognostic implications of delayed diagnosis [26-29].

Overall, this case is clinically relevant because it broadens the recognized spectrum of CPAM presentation. It emphasizes that congenital pulmonary malformations should remain in the differential diagnosis of atypical bilateral apical cystic lesions in adolescents with pneumothorax, and that histopathologic examination may be decisive when imaging findings are inconclusive.

## Conclusions

This case highlights that CPAM can rarely present during adolescence as spontaneous pneumothorax with atypical bilateral apical cystic lesions. Because imaging may not reliably distinguish congenital from acquired cystic lung lesions, histopathologic confirmation remains essential. In symptomatic patients with ambiguous imaging findings, surgical resection may provide both definitive treatment and diagnostic confirmation.
